# Walk-on-Hemispheres first-passage algorithm

**DOI:** 10.1038/s41598-023-28361-1

**Published:** 2023-01-20

**Authors:** Jinseong Son, Dongheyon Shin, Chi-Ok Hwang

**Affiliations:** 1 Division of Liberal Arts and Sciences, Gwangju Institute of Science and Technology, Gwangju Metropolitan City, 61005 South Korea; 2 Gwangju Institute of Science and Technology, Physics Track, Gwangju Metropolitan City, 61005 South Korea; 3 Gwangju Institute of Science and Technology, Electrical Engineering and Computer Science Track, Gwangju Metropolitan City, 61005 South Korea

**Keywords:** Mathematics and computing, Applied mathematics

## Abstract

Due to the isomorphism between an electrostatic problem and the corresponding Brownian diffusion one, the induced charge density on a conducting surface by a charge is isomorphic to the first-passage probability of the diffusion initiated at the location of the charge. Based on the isomorphism, many diffusion algorithms such as “Walk-on-Spheres” (WOS), “Walk-on-Planes” and so on have been developed. Among them, for fast diffusion simulations WOS algorithm is generally applied with an $\varepsilon$-layer, which is used for diffusion convergence on the boundary but induces another error from the $\varepsilon$-layer in addition to the intrinsic Monte Carlo error. However, for a finite flat boundary it is possible to terminate a diffusion process via “Walk-on-Hemispheres” (WOH) algorithm without the $\varepsilon$-layer. In this paper, we implement and demonstrate this algorithm for the induced charge density distribution on parallel infinite planes when a unit charge is between the plates. In addition, we apply it to the mutual capacitance of two circular parallel plates. In both simulations, WOH algorithm shows much better performance than the previous WOS algorithm.

## Introduction

According to the probabilistic potential theory, electrostatic problems can be understood via mathematically isomorphic diffusion ones and vice versa^1,2^. Since the Laplacian operator corresponds to the homogeneous and isotropic Brownian motion, electrostatic problems can be solved via the random walk of the Brownian particle. Accordingly, the induced charge density distribution on a conducting surface by a point charge is equal to the first passage probability distribution of the diffusion initiated at the location of the charge onto the boundary surface.

Based on the isomorphism, fast diffusion Monte Carlo algorithms have been developed^3–10^. In the diffusion Monte Carlo algorithms, “Walk-on-Spheres” (WOS) algorithm^3,9,11^ is generally used to find the first passage location on the absorbing boundary surface. However, for diffusion convergence this algorithm requires a layer to terminate the diffusion process near the boundary surface, which is called the $\varepsilon$-layer^3^. In order to avoid the bias from the layer approximation, Green’s function First-passage (GFFP) algorithms without the layers^5,6^ have been developed.

Among them, “Walk-on-Planes” (WOP) algorithm^7,8^ is employed in cases of infinite flat boundary. However, in the case of parallel infinite boundaries we don’t have a good GFFP algorithm because we have only series solutions or integral representations and don’t have a closed form for the corresponding electrostatic problem. For the parallel infinite boundaries, we have only infinite parallel plates GFFP algorithm^10^. However, the algorithm via the series solution combined with the acceptance-rejection sampling method is somewhat complicated.

In this paper, we implement a new diffusion algorithm called “Walk-on-Hemispheres” (WOH) for a finite flat boundary, which can be also used for the (parallel) finite or infinite planes boundary. In the previous researches of Ermakov^4,12^, he gave mathematical proofs for the transition probabilities regarding the hemisphere geometry based on image charge method and later^4,12,13^ the WOH algorithm was implemented via Von Neumann’s acceptance-rejection method^14^. Here, we implement WOH algorithm by applying a conformal map^15,16^ combined with an acceptance rejection method^14^.

In the following sections, at first the derivation of the WOH sampling formula is given. Next, we demonstrate the algorithm on parallel infinite planes boundary. In addition, the mutual capacitance of two parallel circular plates is computed. Finally, we make a comparison between WOH and WOS algorithms. It is shown that the WOH algorithm is more efficient than the WOS one.

## Results

### “Walk-on-Hemispheres” (WOH) algorithm

**Figure 1:**
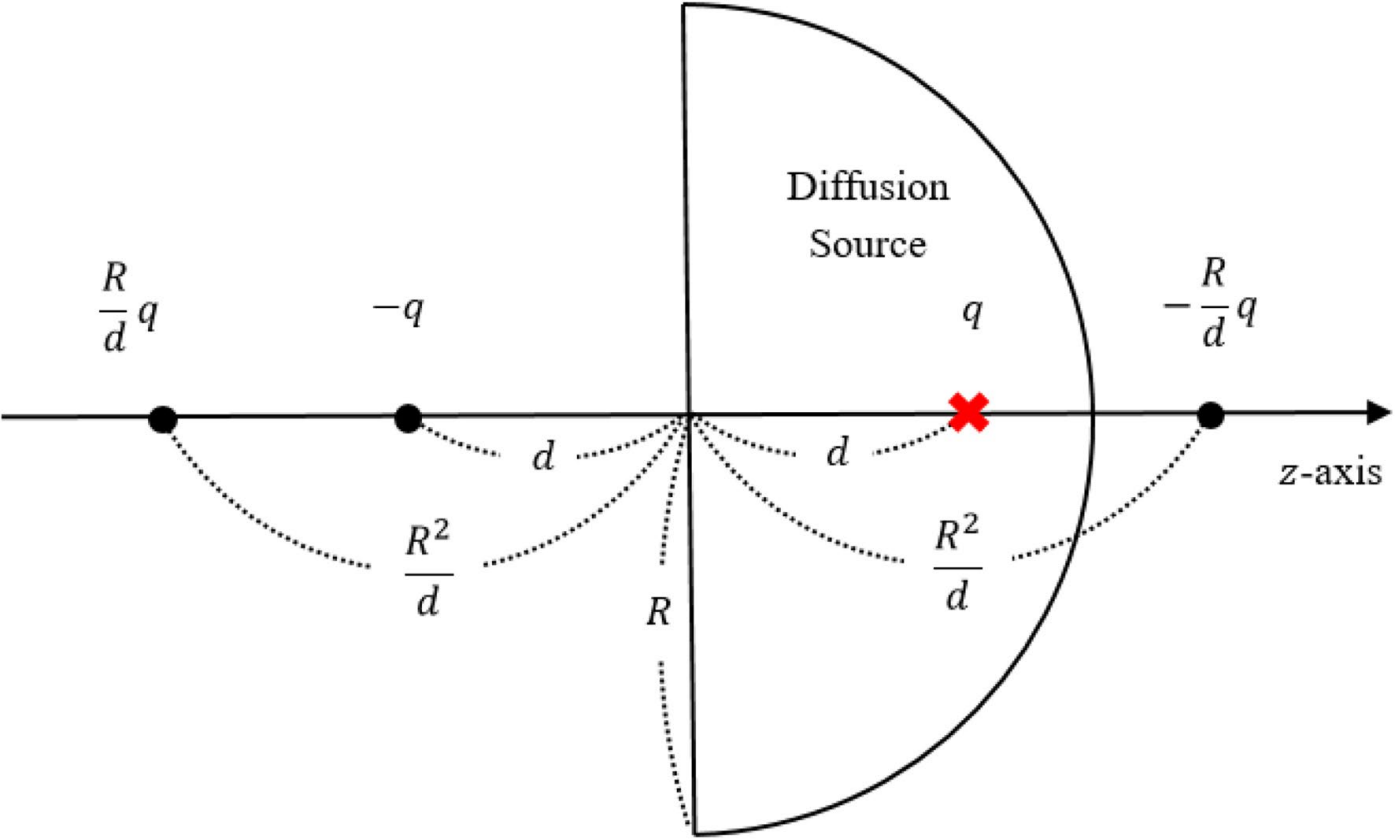
Schematic diagram of WOH algorithm; the radius of the hemisphere *R* and a charge $-q$ at $z=d$ and its three image charges at $z=-d$, $z=-R^2/d$, and $z=R^2/d$ to make the potential zero on the boundary.

In this section, we explain WOH algorithm. In order to obtain the first passage distribution from a diffusion source $\vec {r_{0}}$ (the location of the charge *q*) inside the hemisphere $\Omega$ to a point $\vec{r}$ on its boundary $\partial \Omega$ (see Fig. 1), we need a Green’s function $G(\vec {r_{0}},\vec{r})$ which satisfies the following^17^;

1 $$\begin{aligned} \Delta G(&\vec {r_{0}},\vec{r}) = -\delta (\vec {r_{0}} - \vec{r}), \qquad \text {when} \quad \vec{r} \in \Omega \nonumber \\&G(\vec {r_{0}},\vec{r}) = 0, \qquad \text {when} \quad \vec{r} \in \partial \Omega . \end{aligned}$$

Here, $\Delta$ is the Laplacian operator. Then the normal derivative of $G(\vec {r_{0}},\vec{r})$ on $\partial \Omega$ creates the harmonic measure^18^ and any harmonic function $u(\vec{r})$ in $\Omega$ satisfies the boundary integral equation;

2 $$\begin{aligned} u(\vec {r_0})=\int u(\vec{r}) \frac{\partial G(\vec{r},\vec {r_0})}{\partial {\hat{n}}} \,d S_{\partial \Omega }. \end{aligned}$$

Here, ${\hat{n}}$ is the normal vector inwards the domain. For the required Green’s function $G(\vec {r_{0}},\vec{r})$, the linear combination of electric potentials can be used. To invoke the axial symmetry, we put a negative unit charge and its image charges on *z*-axis as Fig. 1 so that the potential vanishes on the conducting surface. Setting the radius of hemisphere *R* and the height of the source charge from the origin *d*, we obtain the Green’s function in the spherical coordinates $(r,\theta ,\phi )$ (here, $\theta$ from 0 to $\pi$ is the angle from the positive z axis downward) given by

3 $$\begin{aligned} \begin{aligned} G(r,\theta ,\phi ) = \frac{1}{4\pi }&\Bigg ( \Bigg . \frac{1}{ \sqrt{d^{2} + r^{2} - 2d r \cos \theta } } - \frac{R/d}{\sqrt{r^{2} + \frac{R^{4}}{d^{2}} - \frac{2r R^{2} \cos \theta }{d}}} \\&- \frac{1}{\sqrt{d^{2} + r^{2} + 2d r \cos \theta }} + \frac{R/d}{\sqrt{r^{2} + \frac{R^{4}}{d^{2}} + \frac{2r R^{2} \cos \theta }{d}}} \Bigg . \Bigg ). \end{aligned} \end{aligned}$$

Taking the partial derivative of $G\left( r,\theta ,\phi \right)$ with respect to *r*, we get at *R*

4 $$\begin{aligned} \begin{aligned} \frac{\partial G(r,\theta ,\phi )}{\partial r} \Big |_{r=R} = \frac{1}{4\pi }&\Bigg ( \Bigg . \frac{R - d \cos \theta }{(d^2 + R^2 - 2d R \cos \theta )^{3/2}} - \frac{R (d R - R^2 \cos \theta )}{d^2 \left( R^2 + \frac{R^4}{d^2} - \frac{2 R^3 \cos \theta }{d} \right) ^{3/2}} \\&- \frac{R + d \cos \theta }{\left( d^2 + R^2 + 2d R \cos \theta \right) ^{3/2}} + \frac{R\left( d R + R^2 \cos \theta \right) }{d^2 \left( R^2 + \frac{R^4}{d^2} + \frac{2 R^3 \cos \theta }{d} \right) ^{3/2}} \Bigg . \Bigg ). \end{aligned} \end{aligned}$$

Now, let $\gamma$ be the ratio *d*/*R* and the boundary $\partial \Omega = \partial X \cup \partial Y$ ($\partial X$: hemisphere, $\partial Y$: disk) and we integrate over the hemispherical part. The cumulative induced charge density is obtained to be

5 $$\begin{aligned} \Sigma _{X} (\theta ) = \int \limits _{\phi =0}^{\phi =2\pi } \int \limits _{{\tilde{\theta }}=0}^{{\tilde{\theta }}=\theta } \frac{\partial G(r,{\tilde{\theta }},\phi )}{\partial r}\bigg |_{r=R} d S_{\partial \Omega } = \frac{1}{\gamma } - \frac{(1 - \gamma ^2)}{2\gamma } \left( \frac{1}{\sqrt{1+\gamma ^2-2\gamma \cos \theta }} + \frac{1}{\sqrt{1+\gamma ^2+2\gamma \cos \theta }} \right) , \end{aligned}$$

and the total induced charge on the hemisphere becomes

6 $$\begin{aligned} \Sigma _{X} = \int \limits _{\phi =0}^{\phi =2\pi } \int \limits _{{\tilde{\theta }}=0}^{{\tilde{\theta }}=\pi /2} \frac{\partial G(r,{\tilde{\theta }},\phi )}{\partial r}\bigg |_{r=R} d S_{\partial \Omega } = \frac{\sqrt{1+\gamma ^2} - (1-\gamma ^2)}{\gamma \sqrt{1+\gamma ^2}}. \end{aligned}$$

By Eqs. (5) and (6), the conditional cumulative distribution with respect to the azimuthal angle $\theta$ of the first passage location on the hemisphere is given by

7 $$\begin{aligned} P_X(\Theta \le \theta | \vec {r_0} \rightarrow \partial X) = \Sigma _{H} (\theta )/\Sigma _H. \end{aligned}$$

Now, let $u \in U(0, 1)$, $\alpha =1+\gamma ^2$, $\beta =1-\gamma ^2$ and $\mu (u)=(\beta u + (1-u)\sqrt{\alpha }))^2$. Then the inverse transform of the cumulative distribution function is given by

8 $$\begin{aligned} \theta (u) = \cos ^{-1} \left( \frac{\alpha }{8\gamma \mu (u)} \sqrt{16 \mu (u)^2 - 2 \beta ^4 - 8 \beta ^2 \mu (u) - 2 \beta ^3 \sqrt{\beta ^2 + 8 \mu (u)}} \right) \end{aligned}$$

The formula (8) gives the exact sampling on the spherical part of the hemisphere.

**Figure 2:**
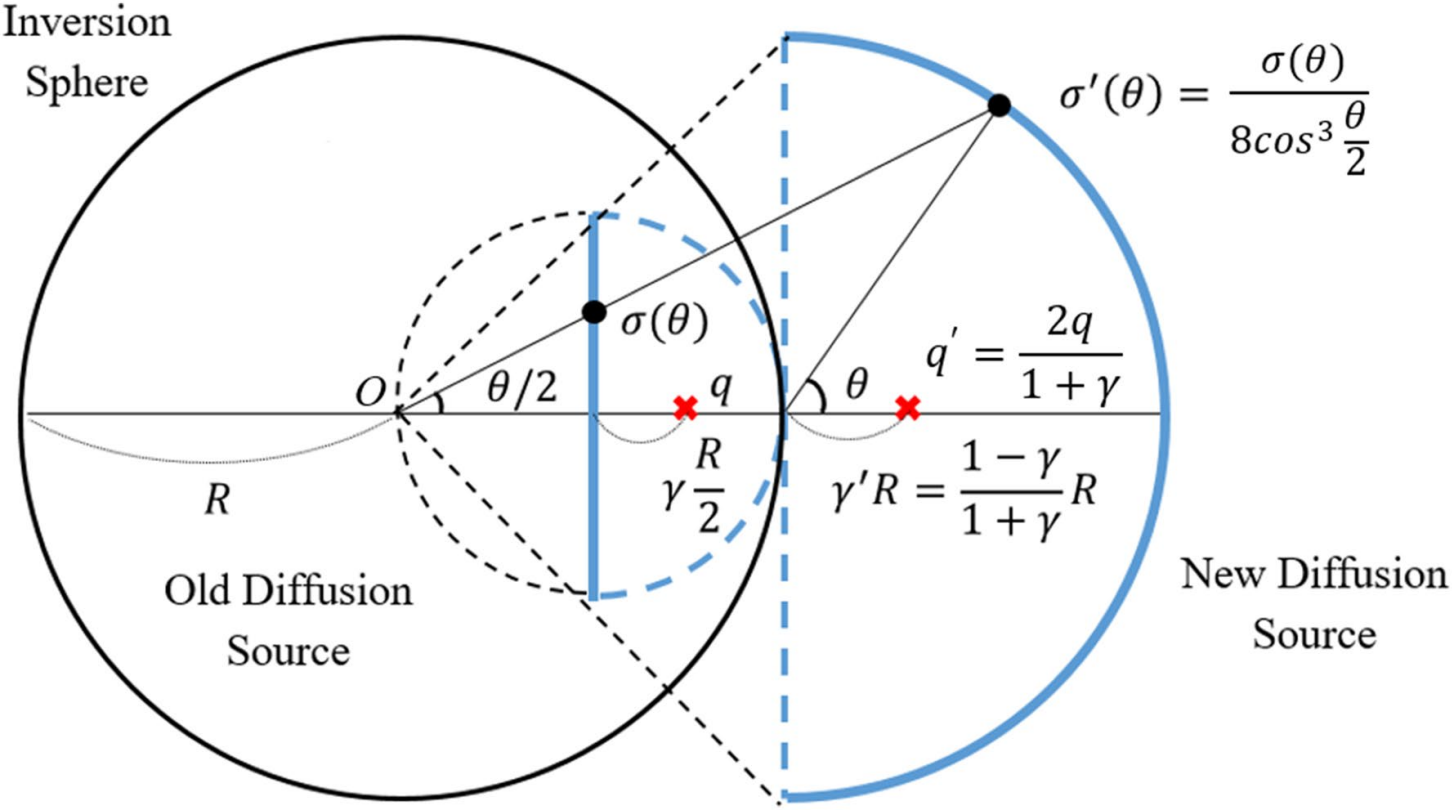
Schematic diagram for the inversion mapping of the disk part of hemisphere.

For sampling on the disk part, because of the complexity of the inverse transformation sampling, we use conformal map^15,16^ to exchange the location of the disk and spherical part as shown in Figure 2.

Let the distance from the origin *O* to the charge *q* and $q^{\prime }$ be *r* and $r^{\prime }$, respectively.

9 $$\begin{aligned} r \rightarrow r^{\prime } = \frac{R^2}{r} \end{aligned}$$

Then, the relation between *r* and $r^{\prime }$ should satisfy the Eq. (9). Thus, the position of the charge $q^{\prime }$ is specified as $r^{\prime } = 2R/(1+\gamma )$. In addition, for convenience of calculation, a variable $\gamma = 2r/R - 1$ and $\gamma ^{\prime } = R/r - 1$ are introduced respectively to represent the distance ratio from the center of the disk and hemisphere.

10 $$\begin{aligned} q^{\prime } = \frac{R}{r} q \end{aligned}$$

In Figure 2, the potential of the transferred charge $q^{\prime }$ is defined by Eq. (10).

11 $$\begin{aligned} \sigma ^{\prime }(r, \theta , \phi ) = \left( \frac{R}{r}\right) ^3 \sigma \left( \frac{R^2}{r}, \theta , \phi \right) \end{aligned}$$

By introducing the azimuthal angle $\theta / 2$ of the inversion sphere by Eq. (11), the induced charge density on the disk surface $\sigma (\theta )$ can be written in terms of $\sigma ^{\prime }(\theta )$.

The relation of the cumulative charges of the disk and the spherical part is given by the following;

12 $$\begin{aligned} \partial _{\theta } \Sigma _{Y} (\theta ; \gamma , q)&= \sigma (\theta ) 2 \pi \left( \frac{R}{2} \right) ^2 \tan \frac{\theta }{2} \sec ^2 \frac{\theta }{2} \nonumber \\&= \sec \left( \frac{\theta }{2} \right) (\sigma ^{\prime } (\theta ) 2 \pi R^2 \sin \theta ) \nonumber \\&= \sec \left( \frac{\theta }{2} \right) \partial _{\theta } \Sigma _X (\theta ;\gamma ^{\prime },q^{\prime }). \end{aligned}$$

For practical use, we first determine which surface the diffusion passes through between the spherical and the disk parts by Eq. (6), the total induced charge on the spherical part. If the spherical part is chosen, then we just sample the angle $\theta$ by Eq.  (8). Otherwise, we compute $\gamma ^{\prime }$ as shown in Fig. 2 and then apply Eq. (8) to sample the angle $\theta$/2. To follow the actual likelihood in Eq. (12), we use the acceptance-rejection method [8] with acceptance probability $\sec (\theta / 2) / \sqrt{2}$.

### Induced charge distribution on parallel infinite planes

**Figure 3:**
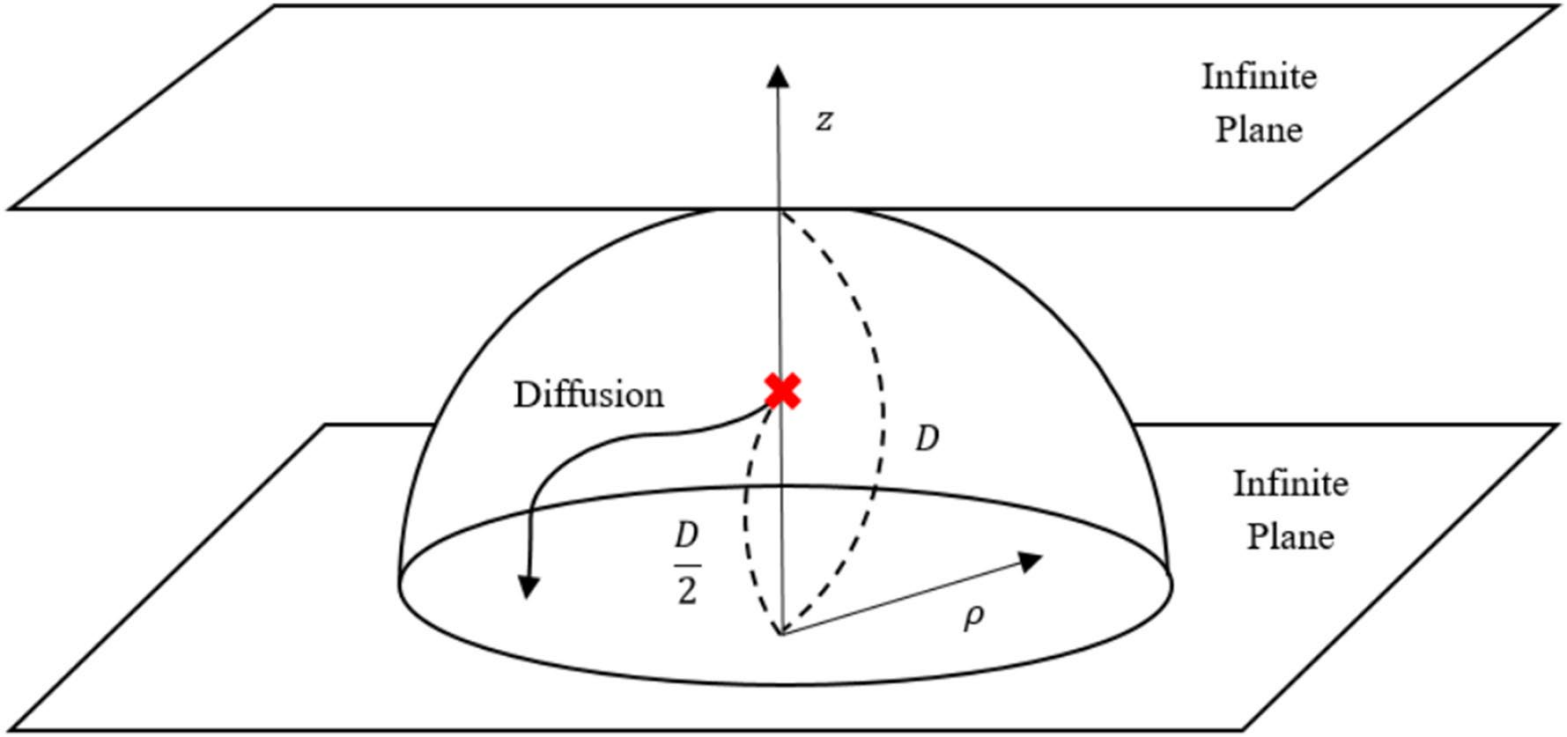
Schematic diagram for two infinite parallel conducting planes and a charge at the middle between the planes; the radius of the hemisphere is *R* (that is *D*), $\rho$ the distance from the center of the hemisphere and the corresponding diffusion starts from the distance *D*/2 from the plane.

When a charge is located between two infinite parallel conductors, the analytic solution for the induced charge density is known as a series solution only^19–21^. For the sample of the corresponding diffusion in diffusion Monte Carlo simulations in this geometry, “Walk-on-Spheres” (WOS) algorithm and recently developed “Infinite Parallel Plates” (IPP) [9] algorithm are available. However, in WOS algorithm the diffusion sample on the parallel boundaries can be biased by the $\varepsilon$-layer if the layer is not thin enough to suppress the error from the layer, which is needed for convergence of the diffusion simulation^22^. In the other IPP algorithm case, we have to use a tabulation and compute the additional terms of series solution whenever the sampling is too close to the rejection criteria so that the algorithm is somewhat complicated^19^.

In this section, the sampling of a diffusion in the parallel planes boundary is performed by WOH algorithm. The initial position of the diffusion is located at the middle of two infinite planes as shown in Fig. 3. For the WOH diffusion, the radius is fixed to *D* and the direction of the disk boundary is toward the plane of the minimum distance. The first-passage distribution of the diffusion simulation is compared to the corresponding electrostatic analytic series solution given below^21^;

13 $$\begin{aligned} \sigma (\rho ) = \frac{1}{\pi } \sum _{n=1}^{\infty } \frac{(-1)^n (1-2n)}{D^2 (4(\rho /D)^2 + (1- 2n)^2)^{3/2}}. \end{aligned}$$

**Figure 4:**
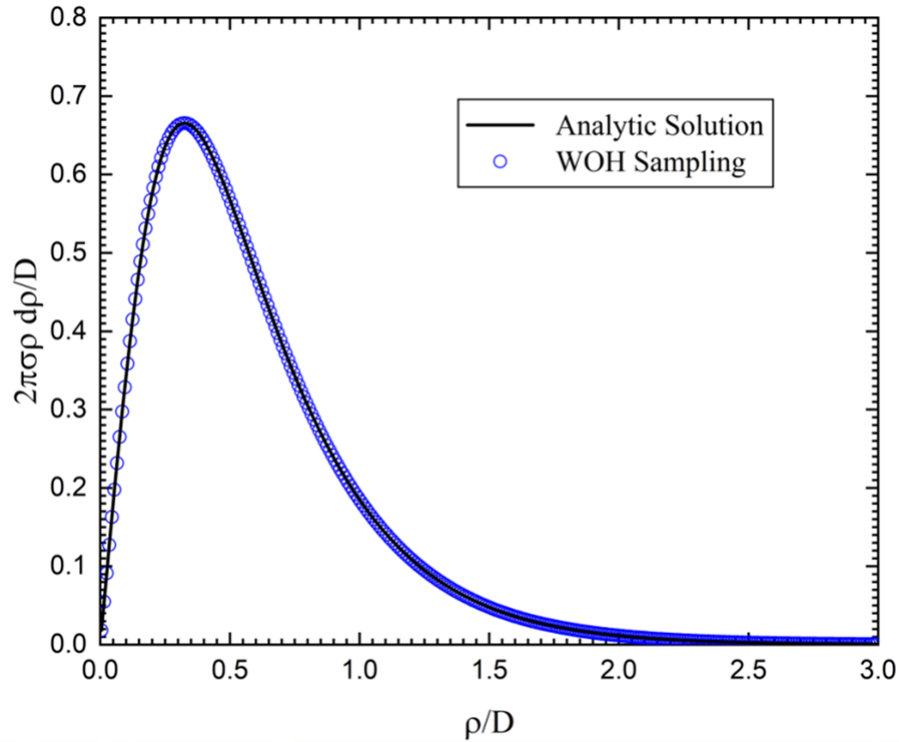
Induced total charge in $\vec {\rho /D}$ by WOH algorithm with 100 independent runs (blue circle) of $10^9$ Monte Carlo steps and its analytic series data (black solid line).

**Figure 5:**
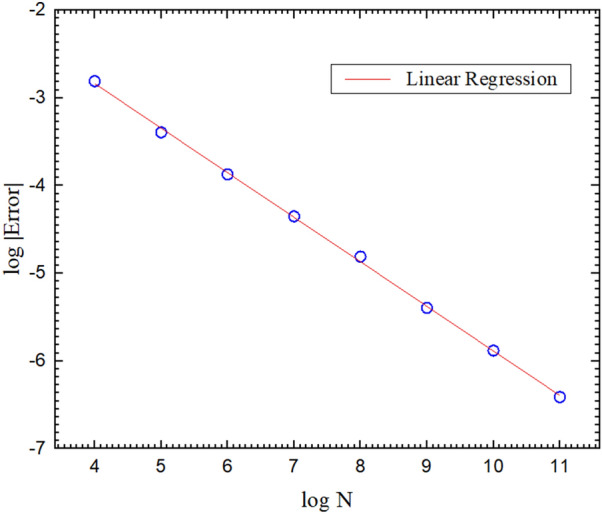
This graph shows the errors (blue circles) of WOH algorithm when we compute the induced charge density on the parallel infinite planes. The red solid line is its linear regression.

The result in Fig. 4 verifies that WOH algorithm provides the correct induced charge distribution. In order to obtain the above result, we performed 100 independent runs of $10^9$ Monte Carlo (MC) steps, that is, via the total number of $10^{11}$ simulated diffusion quasiparticles. The convergence of the Monte Carlo errors is given in Fig. 5. The linear regression has its slope of $- \, 0.49739$ with the correlation coefficient of $- \, 0.99986$. It is noted that all the logarithms used in this paper are the decimal logarithm.

**Figure 6:**
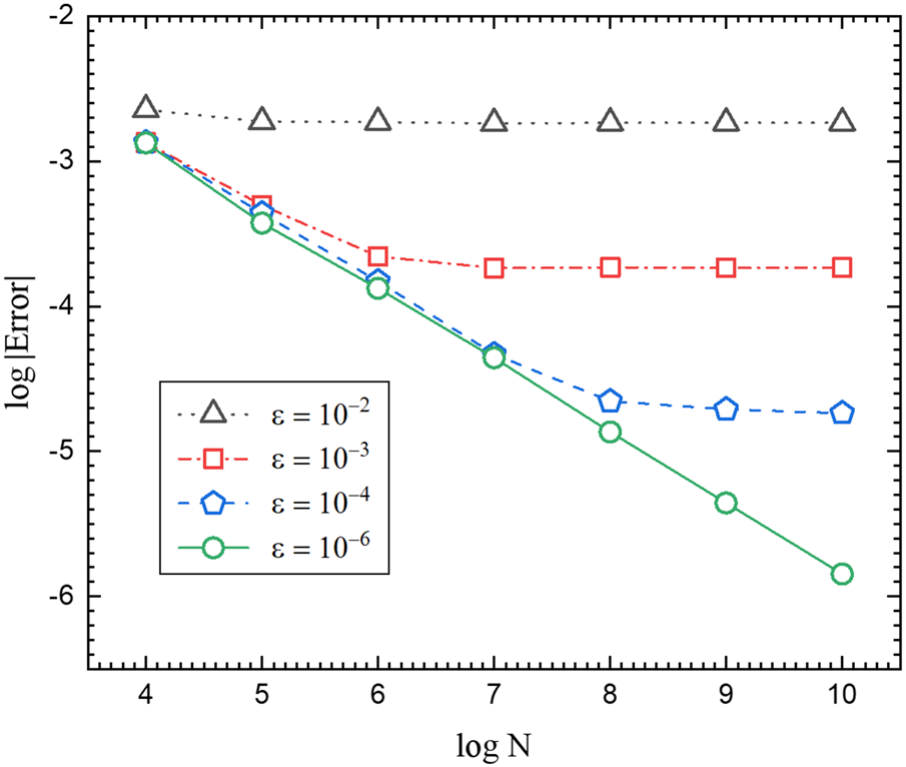
This graph shows the errors of WOS algorithm when we compute the induced charge density on the parallel infinite planes for the cases of $\varepsilon =10^{-2}$, $10^{-3}$, $10^{-4}$ and $10^{-6}$.

In WOS algorithm, for large enough MC steps the Monte Carlo error convergence does not exhibit the linearity due to the error from the $\varepsilon$-layer. If MC steps are large enough, the error from the layer becomes dominant^23,24^. To see the $\varepsilon$-layer error in Fig. 6, we perform the same simulation replacing WOH with the WOS algorithm with various $\varepsilon$-layers. Figure 6 clearly states that the error convergence is hindered by the $\varepsilon$-layer. For the case of $\varepsilon = 10^{-6}$, it seems that the simulation result is not much affected by the layer and the MC intrinsic error is dominant. Although the $\varepsilon$-layer error can be reduced by making the layer smaller, it causes the logarithmic increase of the simulation time^23–25^.

**Figure 7:**
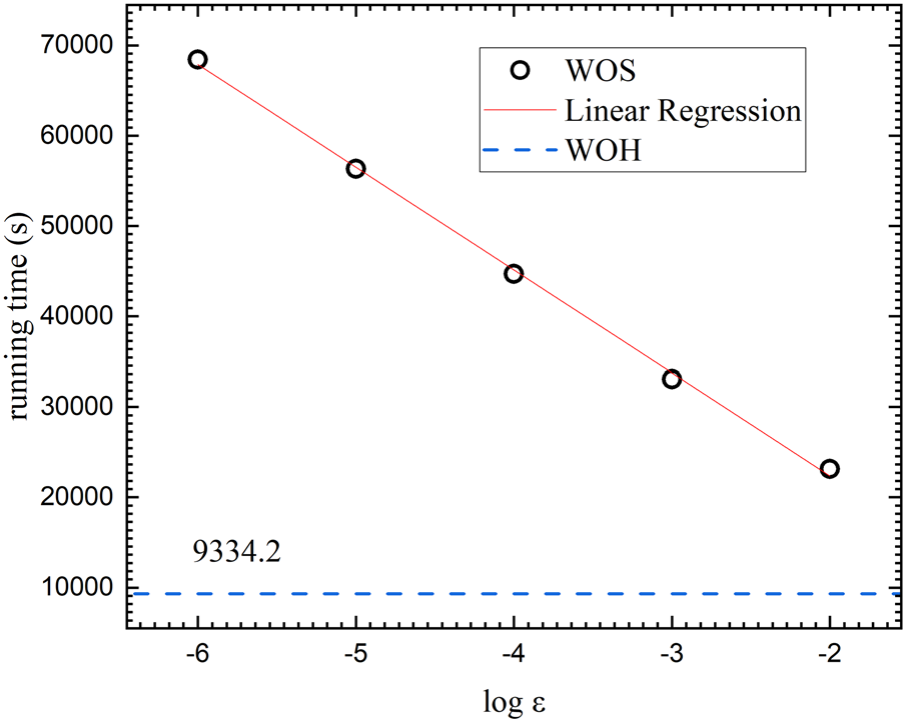
Runtime comparison of WOH algorithm (blue dashed line) and WOS algorithm (black circles); the red solid line is the linear regression of the WOS algorithm. We evaluate the runtimes for the induced charge density on the parallel infinite planes with 100 independent simulations of $10^7$ MC steps.

In addition, in Fig. 7 with the induced charge density on the parallel infinite planes, we investigate the runtimes of the two algorithms, WOH and WOS. The runtime of WOH algorithm is inserted as the blue dotted guideline. The runtimes of WOS algorithm are obtained with $\varepsilon$ from $10^{-6}$ to $10^{-2}$. The linear regression of the WOS algorithm runtimes has its slope around $- \, 10.51$ with the correlation coefficient of $- \, 0.99996$. In the parallel infinite plane simulation, it is clearly verified that the WOH algorithm is much more efficient than the WOS algorithm with any practical choice of $\varepsilon$-layer.

### Mutual capacitance of two parallel circular plates

In this section, we demonstrate WOH algorithm for the mutual capacitance of two parallel circular plates with various radii and compare the performances with the WOS algorithm.

The mutual capacitances $C_{ij}$ of multi-conductors are represented in the matrix form which shows the relations between the total charge $Q_i$ on the *i*-th conductor and the voltages $V_j$ on the *j*-th conductor among *N* conductors;

14 $$\begin{aligned} Q_i = \sum _{j=1}^{N} C_{ij} V_{j}. \end{aligned}$$

**Figure 8:**
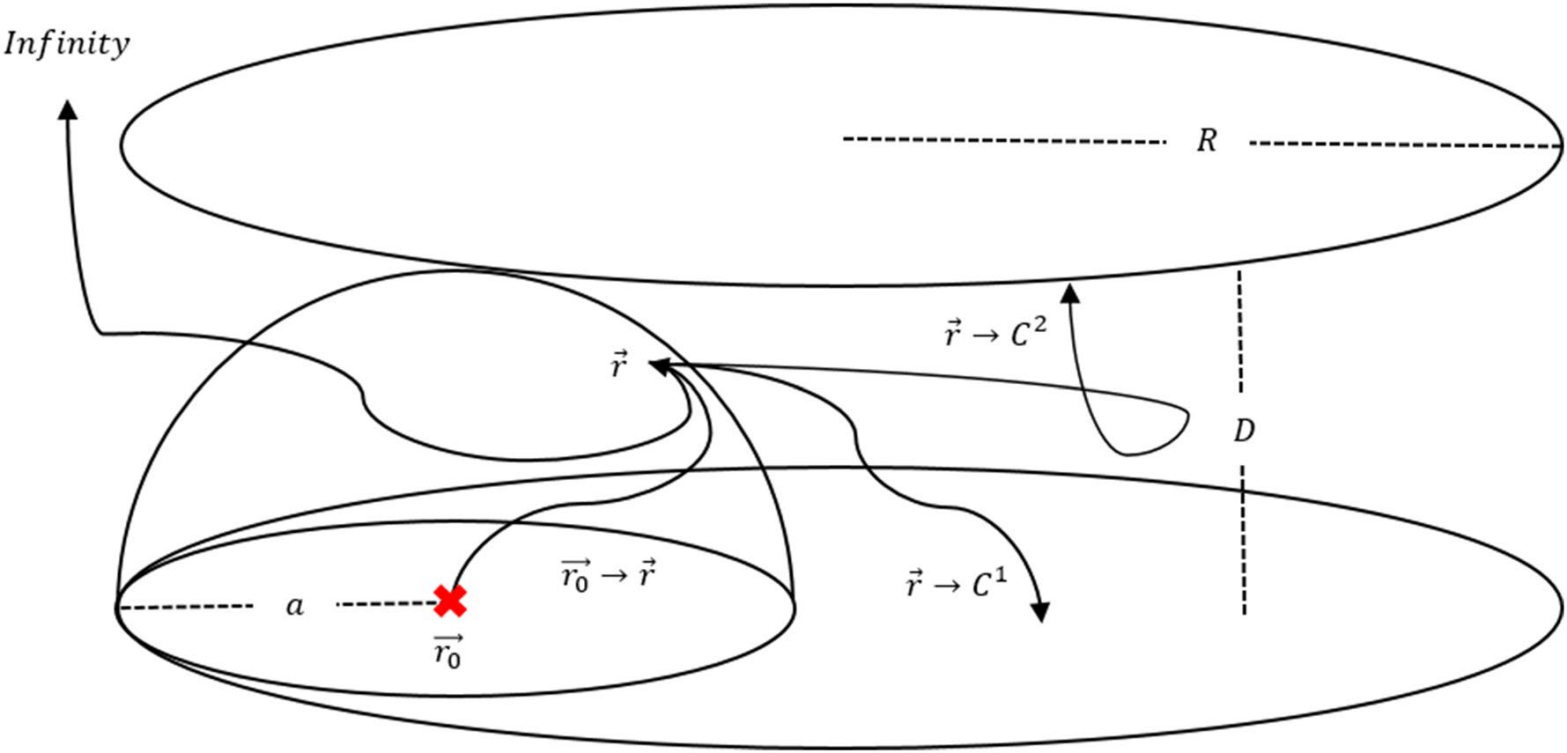
Schematic diagram of the last passage algorithm^26,27^ for the mutual capacitance of the parallel circular plates.

Here in this paper, as in Fig. 8 we have two conductors only and so $N=2$. We compute the total charge on the *i*-th conductor by integrating the surface charge density on it via the last passage algorithm^26,27^, which can compute the charge density at a specific point on a conductor. The surface charge at $\vec {r_{0}}$ is given in terms of the last passage Green’s function, $g(\vec{r},\vec {r_0})$, of radius *a* of the last-passage hemisphere and the probability, $P(\vec{r} \rightarrow \infty )$, of going to infinity of the diffusion which is initiated at $\vec {r_{0}}$.

15 $$\begin{aligned} \sigma (\vec {r_0}) =&\frac{1}{4 \pi } \oint g(\vec{r},\vec {r_0})P(\vec{r} \rightarrow \infty ) d S_{\partial \Omega }, \nonumber \\ g(\vec{r}, \vec {r_0})&= \frac{3 \cos \theta }{2 \pi a^3}. \end{aligned}$$

Here, the surface integration is over the last-passage hemisphere, $\partial \Omega$.

When the *i*-th conductor $C_i$ has unit voltage and the others are grounded, the surface charge density $\sigma _{ii}$ on $C_i$ and $\sigma _{ij}$ on $C_j$ are represented by;

16 $$\begin{aligned} \sigma _{ii}(\vec {r_0})&= \frac{1}{4 \pi } \oint g(\vec{r},\vec {r_0}) [1 - P(\vec{r} \rightarrow C_i)] d S_{\partial \Omega }, \nonumber \\ \sigma _{ij}(&\vec {r_0}) = \frac{1}{4 \pi } \oint g(\vec{r},\vec {r_0}) [P(\vec{r} \rightarrow C_j)] d S_{\partial \Omega }, \end{aligned}$$

where the probabilities, $P(\vec{r} \rightarrow C_i)$ and $P(\vec{r} \rightarrow C_j)$, are of going to capacitor *i* and *j* of the diffusions which are initiated at $\vec {r_{0}}$ respectively.

For the two circular plate conductor, the elements of capacitance matrix $C_{11}$ and $C_{12}$ are given by integrating the corresponding charge density over the conducting surfaces [10].

17 $$\begin{aligned}{} & {} C_{11} = \oint \sigma _{11} \,dS_{\partial \Omega } \nonumber \\{} & {} C_{12} = \oint \sigma _{12} \,dS_{\partial \Omega } \end{aligned}$$

Let the distance between the two capacitors *D* and their radius *R* as shown in Fig. 8. We compute the mutual capacitance for various geometries by changing the ratio *R*/*D* from 0.1 to $10^3$. The simulation results are shown in the Table 1 from 100 independent runs of $10^{10}$ Monte Carlo steps. The analytic solutions in Table I are taken from the references^27,28^.

**Table 1:** Mutual capacitance comparison between the analytic and simulated results in dimensionless form, $(\pi /R)C$; *R*/*D* is the ratio of the radius of the circular plate and the separation between the plate.

R/D	$\mathbf {C_{11} + C_{12}}$		$\mathbf {C_{11} + C_{12}}$	
	Analytic	Simulation	Analytic	Simulation
1000	787.8567	787.8549	0.50070	0.50057
100	80.4345	80.4346	0.50537	0.50514
10	9.2331	9.2323	0.53588	0.53577
1	1.8208	1.8203	0.69120	0.69101
0.1	1.0675	1.0675	0.94051	0.94067

The runtime comparison of WOH algorithm with WOS one for the mutual capacitances of the two parallel circular plates from 100 independent simulations of $10^7$ MC steps is given in Table 2 with respect to the geometries given by the ratios of the radius to the separation *R*/*D* from 0.1 to $10^3$. With the WOS algorithm, we used the $\varepsilon$-layers, $10^{-2}$, $10^{-3}$, $10^{-4}$, $10^{-5}$ and $10^{-6}$. The runtimes of WOS are not significantly changed with respect to the geometry. With WOH algorithm, the runtime increases as the *R*/*D* ratio decreases and is less than WOS algorithm except the case of $R/D=0.1$ and $\varepsilon =10^{-2}$.

**Table 2:** Runtime (in seconds) comparison of WOH algorithm with WOS one; for the mutual capacitance of the two parallel circular plates we use 100 independent simulations of $10^{10}$ MC steps.

$$\mathbf {\quad CPU~time~per~run~(s)}$$						
R/D	WOH	WOS $(\varepsilon = 10^{-2})$	WOS$(\varepsilon = 10^{-3})$	WOS$(\varepsilon = 10^{-4})$	WOS$(\varepsilon = 10^{-5})$	WOS $(\varepsilon = 10^{-6})$
1000	10,616	24,498	32,126	39,541	47,252	57,021
100	11,185	24,673	32,510	40,176	47,940	55,760
10	14,762	27,551	34,891	42,991	50,761	58,602
1	26,311	33,424	40,851	48,182	56,289	63,020
0.1	33,440	31,490	38,666	45,400	51,982	59,198

*R*/*D* is the ratio of the radius of the circular plate and the separation between the plates.

The runtime data are obtained from 100 independent runs of $10^10$ Monte Carlo steps. All computations were performed on a MPI PC cluster (15 nodes, 160 cores with 2.40 GHz and 120 cores with 2.10 GHz) with scalable parallel random number generator (SPRNG)^29^.

## Discussion

Due to the isomorphism between a Brownian diffusion problem and the corresponding electrostatic one, the induced charge density on a conducting surface by a charge inside the boundary can be obtained by the first-passage probability of the diffusion initiated from the charge location and vice versa. In this isomorphism, an absorbing boundary surface in the diffusion problem corresponds to the conducting surface in the electrostatic one, a diffusion-starting position to the charge location, first-passage distribution on the absorbing boundary to the induced charge distribution on the conducting surface respectively.

Based on the isomorphism, fast diffusion Monte Carlo algorithms have been developed, such as “Walk-on-Spheres” (WOS) algorithm, “Walk-on-Planes” (WOP) algorithm, “Walk-on-Hemispheres” (WOH) algorithm, “Walk-on-Cubes” algorithm and so on^3,5–8,10,30^. Among them, the simplest WOS algorithm is generally used with an $\varepsilon$-layer due to the fact that WOS algorithm can be used in any geometrical boundaries. However, it should be noted that the $\varepsilon$-layer for the convergence of diffusion induces an error^23,24^. Without the $\varepsilon$-layer, for an infinite flat boundary it is possible to terminate the diffusion process via WOP algorithm^7,8^. For a finite flat boundary or in two-plate between boundaries, WOP algorithm can not be used and WOH one is very plausible. In the previous works^4,12,13^, WOH algorithm was implemented via Von Neumann’s accecptance-rejection method^14^. In this paper, via a conformal map^15,16^ combined with an accecptance-rejection method^14^ we implement and demonstrate the WOH algorithm for the induced charge density distribution on parallel infinite conducting plates when a unit charge is between the plates and apply it to the mutual capacitance of two circular parallel plates. In both simulations, WOH algorithm shows much better performance than the WOS algorithm.

Diffusion Monte Carlo algorithms have been mainly applied for extracting mutual capacitances for a system of conductors,^12,13,31,32^. In semiconductor industry, a powerful commercial 3D CAD tool, *QuickCap*$^{\hbox {TM}}$ has been used^33^. It is emphasized that WOH algorithm can be used for the cases of diffusion near to a finite plane boundary or the one surrounded by two-plane between boundaries.

## Data Availability

The datasets used and/or analysed during the current study are available from the corresponding author on reasonable request.
